# Food Additives Derived from Fruits and Vegetables for Sustainable Animal Production and Their Impact in Latin America: An Alternative to the Use of Antibiotics

**DOI:** 10.3390/foods13182921

**Published:** 2024-09-15

**Authors:** Marina Golowczyc, Andrea Gomez-Zavaglia

**Affiliations:** Center for Research and Development in Food Cryotechnology (CIDCA), CCT-CONICET La Plata, La Plata RA1900, Argentina; mgolowczyc@yahoo.com.ar

**Keywords:** fruit and vegetable co-products, livestock, food safety, sustainability, regulatory authorities

## Abstract

The production of healthy animal-derived food entails the effective control of foodborne pathogens and strategies to mitigate microbial threats during rearing. Antibiotics have been traditionally employed in animal farming to manage bacterial infections. However, the prohibition of antibiotic growth promoters in livestock farming has brought significant changes in animal production practices. Although antibiotics are now restricted to treating and preventing bacterial infections, their overuse has caused serious public health issues, including antibiotic resistance and the presence of antibiotic residues in food and wastewater. Therefore, sustainable animal production is crucial in reducing the spread of antibiotic-resistant bacteria. Annually, 40–50% of fruit and vegetable production is discarded worldwide. These discards present significant potential for extracting value-added ingredients, which can reduce costs, decrease waste, and enhance the food economy. This review highlights the negative impacts of antibiotic use in livestock farming and stresses the importance of analyzing the challenges and safety concerns of extracting value-added ingredients from fruit and vegetable co-products at an industrial scale. It also explores the current trends in reducing antibiotic use in livestock, with a focus on Latin American contexts. Finally, the suitability of using value-added ingredients derived from fruit and vegetable co-products for animal feeds is also discussed.

## 1. Introduction

The utilization of antibiotics as growth promoters in animal production, particularly within intensive farming systems for pigs, chickens, and cows, has been a longstanding practice [1,2]. Sub-therapeutic administration of antibiotics at low doses effectively maintains a controlled level of pathogenic bacteria, preventing their detrimental impact on the health and development of animals. This, in turn, stimulates growth, enhances feed efficiency, and ultimately leads to increased production of meat, milk, and eggs. However, the utilization of antibiotics promotes the selection of resistant bacteria, which can be transmitted to humans through contact with animals or by consuming raw fruits and vegetables that harbor these pathogens. This situation poses a significant threat to public health, as individuals infected with antibiotic-resistant pathogens face limited therapeutic options for certain treatments, thereby reducing the likelihood of successfully combating the infection.

Recent studies have projected that the excessive use of antibiotics in agro-veterinary activities will contribute to a staggering increase in deaths related to antimicrobial resistance. It is estimated that this impact could result in approximately 10 million deaths annually by the year 2050 [3]. In light of these emerging concerns, there has been a growing focus on the utilization of alternative strategies for promoting animal growth and health. These strategies include the use of probiotics, prebiotics, and plant-based feed additives that can enhance gut health and boost the immune system of animals without the risk of promoting antibiotic resistance [4]. This sustainable animal production certainly contributes to minimizing the spread of antibiotic-resistant bacteria.

Every year, approximately one-third of the global food production intended for human consumption, equivalent to about 1.3 billion tones, goes to waste. In terms of value, this amounts to around USD 990 billion, encompassing losses incurred during food production and food processing [5]. Food processing residues or co-products typically arise from the transformation of raw materials into food products [6,7,8]. However, these co-products face challenges in terms of commercial utilization due to their elevated water content (a_w_, 0.70–0.95) that makes them susceptible to pathogen growth, their trend for rapid auto-oxidation when containing high levels of fat, and their pronounced enzymatic activity that accelerates spoilage processes [9,10]. The economic and legal constraints associated with drying, storage, transportation, and disposal of these co-products further exacerbate the problem [11]. In this scenario, the fruit and vegetable supply chain is one of the main contributors to the generation of these co-products [12,13,14], with about 40–50% of their production being discarded [15]. Peel fractions, seeds, pits, pulps, pomace and leaves are the main co-products [16].

The co-products arising from fruits and vegetables pose a significant environmental challenge as they are often disposed of in landfills or bodies of water, contributing to waterway blockage and heightened organic pollution [17]. In many developing nations, a shift in agricultural practices is occurring, favoring more profitable fruit and vegetable cultivation over traditional cereals [18]. This transition is anticipated to lead to substantial quantities of fruit and vegetable co-products in the future. Recycling these materials and reintegrating them into the food chain by converting them into animal feed appears to be a suitable mitigation strategy. Beyond their use as feed constituents, some of these products also contain beneficial bioactive compounds that can be harnessed as phytogenic additives that contribute to animal well-being.

This review seeks to highlight the detrimental effects of antibiotic use in livestock farming. It also explores the extraction and utilization of value-added ingredients from fruit and vegetable co-products, their bioactive properties as feed additives (antimicrobial, antioxidant, prebiotic effects), their impact on animal production, and their potential as alternatives to traditional antibiotics in livestock. Finally, it examines the specific application of these value-added ingredients in the Latin American context.

## 2. Risks of Antibiotics in Livestock

**Figure 1:**
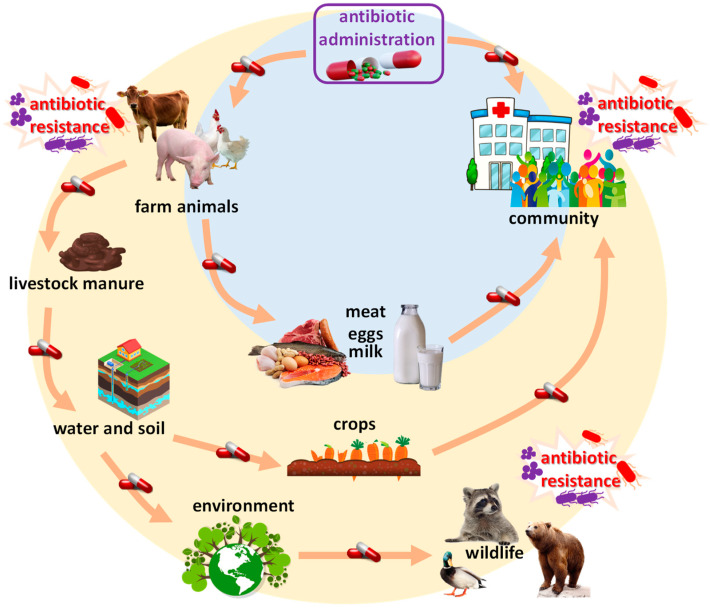
Schematic representation of the food chain risks associated with antibiotic use in animal production and environmental dissemination.

The use of antibiotics in farmed animals has become a widespread practice globally, primarily aimed at promoting growth and preventing diseases in livestock. Figure 1 illustrates the interconnected pathways of antibiotic use in animal production, human communities, and the environment, emphasizing the emergence of antibiotic resistance. Human communities play a crucial role in both the production and use of antibiotics, from healthcare settings to our homes. Unfortunately, a significant portion of antibiotic waste finds its way into sewage systems, contaminating our water, soil, and the environment as a whole. This environmental exposure to antibiotics can lead to the development of antibiotic resistance in bacteria within humans and wildlife. Antibiotic-resistant bacteria can then spread through a variety of pathways, including direct infections, contaminated food sources, and even environmental exposure, ultimately posing a significant threat to public health. In many countries, antibiotics are administered to animals in sub-therapeutic doses as a preventive measure, even in the absence of clinical disease [1]. This practice is especially prevalent in intensive farming systems, where large numbers of animals are kept in confined spaces, increasing the risk of disease outbreaks. The widespread use of antibiotics has raised significant concerns about the potential risks, particularly the development and spread of antibiotic-resistant bacteria [19].

The presence of antibiotic residues in food and the environment is another critical issue linked to the use of antibiotics in farmed animals. When animals are treated with antibiotics, traces of these drugs can remain in their tissues, milk, and eggs. If proper withdrawal periods are not observed, these residues can end up in the food supply, posing health risks to consumers [20,21,22,23,24,25]. Consumption of food products containing antibiotic residues can lead to allergic reactions and contribute to the development of antibiotic-resistant bacteria in the human gut [26,27]. This problem is also exacerbated by international trade and travel, which facilitate the global dissemination of resistant pathogens [28,29].

Moreover, antibiotics excreted by animals can contaminate the environment. Manure and wastewater from farms often contain significant amounts of antibiotics, which can leach into soil and water bodies. This environmental contamination can adversely affect wildlife and contribute to the proliferation of antibiotic-resistant bacteria in natural ecosystems [30,31]. These resistant bacteria can be transferred to humans through different pathways, including the consumption of contaminated water or crops irrigated with contaminated water.

This information highlights the widespread implications of antibiotic use in farmed animals, as antibiotic-resistant bacteria originating in livestock can be transmitted to humans through diverse pathways. Considering these concerns, the World Health Organization (WHO) has recognized antimicrobial resistance as one of the top ten global public health threats and has called for coordinated efforts to address the issue at both national and international levels [32].

In Europe, the use of antibiotics in farm animals has been subject to stringent regulations. The European Union (EU) has taken proactive steps to mitigate the risks associated with the use of antibiotics in agriculture, including the prohibition of their use as growth promoters since 2006 [33]. Moreover, the EU’s recent Veterinary Medicines Regulation, which came into effect in January 2023, further restricts the prophylactic use of antibiotics in livestock [34]. These measures aim to reduce the occurrence of antibiotic resistance and protect public health. However, challenges remain, as compliance with these regulations varies across member states, and the illegal use of antibiotics still persists in some areas [35].

In contrast, Latin America presents a different scenario regarding antibiotic use in animal farming. Many countries in the region lack comprehensive regulations and enforcement mechanisms to control their use. As a result, antibiotics are often readily available and used extensively, not only for therapeutic purposes but also as growth promoters [36]. This uncontrolled use poses significant risks, including the rapid emergence and spread of antibiotic-resistant bacteria. Although efforts to tackle this issue in Latin America are underway, with several countries working towards implementing stricter regulations and raising awareness about the responsible use of antibiotics in agriculture, the problem remains unresolved, and much work still needs to be done [37].

To mitigate the risks associated with antibiotic use in farmed animals, it is essential to adopt a multifaceted approach. This includes implementing stringent regulations to control the use of antibiotics, promoting good animal husbandry practices, and investing in research to develop alternative strategies for disease prevention and animal growth promotion. Furthermore, raising awareness among farmers, veterinarians, and the general public about the serious problems arising from antibiotic resistance is crucial for safeguarding public health and combating the growing threat of antibiotic resistance.

## 3. Feed Additives as Antibiotics Alternatives

Fruit and vegetable co-products account for approximately 16% of total food discards and contribute about 6% to global greenhouse gas emissions. According to the Food and Agriculture Organization (FAO), fruits and vegetables represent the largest group of discarded food items, exacerbating the issue of their management and environmental impact [38]. These problems occur in both developed and developing countries. In developed countries, high consumer standards and over-purchasing result in a significant amount of these discards, commonly referred to as ‘waste’. In developing countries, inadequate postharvest, storage, and transportation infrastructure are the primary contributors, leading to what is properly termed ‘losses’ [39].

**Figure 2:**
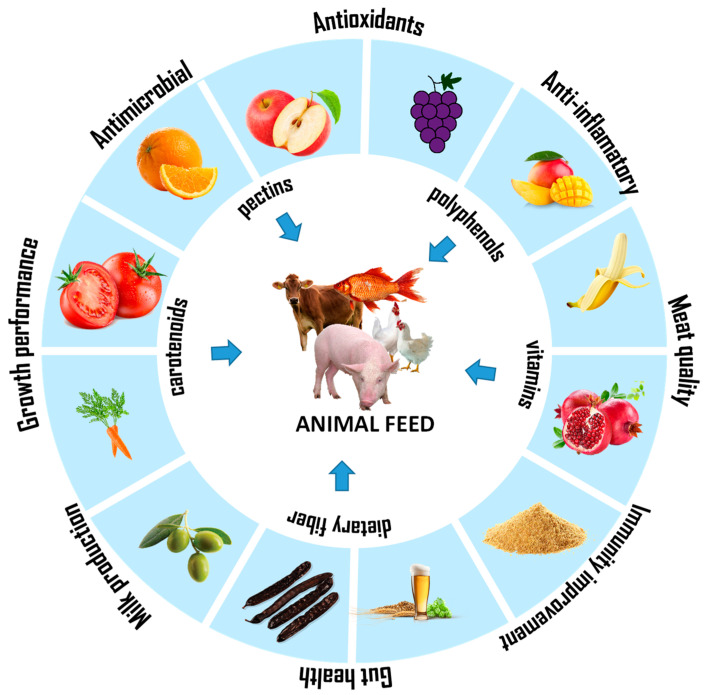
Potential nutritional benefits of fruit and vegetable loss and waste suitable for animal production.

The diversity of fruit and horticultural production, along with the surplus of certain fruits or vegetables during peak production months, presents unique opportunities to add value and reduce loss and waste. These plant materials are rich in phytogenics, that is, bioactive compounds [39] that offer significant market potential as alternatives to antibiotics in livestock farming, enhancing the animals’ health and growth performance (Figure 2). Horticultural co-products represent a valuable resource for animal production, as they are rich in dietary fiber, minerals, vitamins, and phytochemicals, providing potential nutritional advantages for livestock. Incorporating these co-products into animal diets can positively impact animal health, enhance productive performance, and contribute to environmental sustainability. These additives have been successfully incorporated into livestock feed formulations and into aquaculture, and are now under evaluation for their efficacy in pets [40,41]. They are recognized for their bioactive properties, including antioxidant, antimicrobial, anticarcinogenic, analgesic, insecticidal, antiparasitic, and growth-promoting effects, as well as for stimulating bile secretion, enhancing appetite, and elevating digestive enzyme activity [42].

Table 1 provides an overview of selected vegetable and fruit waste and loss products, highlighting their associated bioactive compounds and their potential as sources of phytogenic feed additives for livestock. It also considers the challenges related to their utilization in feed production. The use of fruit and vegetable co-products as feed additives provides dual benefits: it mitigates the environmental impact of food loss and waste and enhances the sustainability of animal production systems. The incorporation of these inhibitory compounds into animal feed allows farmers to reduce their dependence on traditional antibiotics, which in turn addresses public health concerns related to antibiotic resistance while enhancing the efficiency and productivity of farming practices. This innovative approach not only helps in managing food waste but also contributes to the development of a more sustainable and health-conscious animal production industry.

**Table 1:** Commonly used plant loss and waste as feed additives or ingredients in the animal feed industry.

Plant Waste/ Co-Product	Animal Feed Application	Bioactive Components	Benefits	Challenges	References
**Soybean meal**	Poultry, swine, ruminants	Isoflavones (genistein, daidzein), saponins, fiber	Source of protein and amino acids; enhances animal growth	May contain anti-nutritional factors (e.g., trypsin inhibitors)	[43,44]
**Citrus peel and pulp**	Poultry, swine, ruminants	Hesperidin, naringin, limonoids, flavonoids	Rich in vitamin C, fiber and antioxidants; improves animal immune response	High in moisture; can be susceptible to spoilage.	[45,46]
**Apple pomace**	Poultry, swine	Pectins, procyanidins, quercetin, flavonoids	High in fiber; improves gut health and feed palatability	May contain high levels of sugar, leading to digestive issues	[47,48]
**Brewer’s grains**	Cattle, pigs	β-Glucans, fiber, protein, minerals (P, K, Mg)	High in protein, fiber, and B vitamins; improves milk production in dairy cows	May have high levels of fiber; can be prone to spoilage	[49,50]
**Potato peel**	Poultry, swine	Fiber, potassium, vitamin C, polyphenols, carotenoids	High in starch and fiber; can replace a portion of grain in poultry diets; antioxidant capacity	May contain high levels of moisture and sugars	[51]
**Olive pomace**	Poultry, swine	Polyphenols (oleuropein, hydroxytyrosol), fiber, antioxidants	Rich in polyphenols and antioxidants; improves animal immune response	May contain high levels of fat; can be susceptible to spoilage	[7]
**Grape pomace and seed extract**	Broilers, duck, poultry, pig	Resveratrol, anthocyanins, proanthocyanidin, flavonoids	Antioxidant capacity; growth performance; improves immunity and meat quality	Solubility of grape extracts; toxicities associated with the high and continuous consumption	[52,53,54,55,56]
**Mango seeds and peel**	Poultry	Polyphenols (mangiferin, catechin), fiber,	Growth performance; antioxidant capacity	Presence of anti-nutritional compounds	[57]
**Pomegranate peels and pulp**	Poultry, fish	Punicalagin, punicic acid, ellagic acid, antioxidants, high amounts of phenolic acids, flavonoids and tannins	Increases physicochemical and microbiological stability of meat Enhances immunity; anti-inflammatory and gut health benefits;antioxidant capacity	Complex extraction methods;antinutritional effects and palatability	[58,59,60]
**Carrot peel**	Laying hens, poultry, swine	β-Carotene and α-carotene, vitamin C, fiber	Improves digestion, health and immune function;antioxidant capacity	High moisture content; presence of potentially toxic compounds (nitrates and solanines)	[61,62]
**Banana peel**	Ruminants, broilers	Potassium, fiber, vitamins C and B	Antioxidant capacity; gut health benefits	High moisture content requires careful processing and handling to prevent spoilage	[63,64]
**Tomato seed and peel**	Cattle, poultry, sheep and goats, and swine	Lycopene, flavonoids, phenolic compound, vitamins C and E	Enhanced immune function; anti-inflammatory effects; improves meat quality; fiber source	Palatability; complex processing methods; antinutritional factors (lectins)	[65]

## 4. Relevant Bioactive Properties of Fruit and Vegetable Loss and Waste Supporting Their Potential as Antibiotic Alternatives

Different compounds present in fruit and vegetable loss and waste are responsible for their established bioactivities (Table 1, Figure 2) [7,66,67,68]. At the physiological level, the biological activities of these compounds contribute to different plant functions, including their defense mechanisms and ecological interactions. These molecules can act as attractants in seed dispersal, protectants against ultraviolet radiation, and restraints to herbivorous animals by imparting bitter, astringent, or unpleasant flavors [69]. There are two major groups of bioactive compounds: essential and non-essential. The former comprises mostly vitamins and minerals, essential to preventing diseases and to maintaining specific biochemical processes in the consumer [70,71]. In turn, non-essential bioactive compounds comprise metabolites such as phenolics, carotenoids, phytosterols, saponins, essential oils, and phytic acids, which allow the maintenance of optimal cellular health, leading to an improvement in longevity [69,72]. Phenolics and carotenoids are the most widely distributed bioactive compounds available in fruit and vegetable loss and waste [69].

Another aspect that should not be overlooked is that the composition of processed fruit waste varies greatly depending on the type of fruit and the primary component of the waste [73]. For example, waste predominantly composed of whole fruits will yield substantial quantities of monosaccharides and disaccharides. Banana waste, a co-product resulting from export regulations, constitutes 5% to 30% of harvested bananas [64]. Conversely, waste mainly consisting of peels, shells, and seeds will largely consist of structural polysaccharides [74]. This variation also applies to the essential and non-essential compounds present in the waste, and consequently, to their bioactivities.

The main bioactive properties of these compounds, which are associated with their potential as antibiotic alternatives, are detailed in the following subsections.

### 4.1. Antimicrobial Effects

The antimicrobial agents derived from plant secondary metabolites possess the potential for application in intensively farmed animals, enhancing animal health, productivity, and the overall meat quality of food animals [75]. Phytochemicals such as flavonoids, tannins, saponins, and essential oils exhibit broad-spectrum antimicrobial activity against a variety of pathogens including bacteria, fungi, and viruses. These compounds interfere with microbial cell walls, disrupt membrane integrity, inhibit protein synthesis, and impede the function of microbial enzymes, effectively reducing the microbial load and preventing infections [39,76].

Although this review explores horticultural co-products as potential alternatives to antibiotics in animal production, there is, indeed, a need to delve deeper into the specific active ingredients responsible for their beneficial effects. The antimicrobial, antioxidant, and prebiotic properties of these co-products are primarily attributed to bioactive compounds such as polyphenols, flavonoids, carotenoids, and essential oils, among others [77,78]. Understanding the precise mechanisms of action of these compounds is crucial to optimizing their use as feed additives. For instance, flavonoids and tannins disrupt microbial cell walls, while saponins inhibit protein synthesis, and polyphenols provide antioxidant effects that support gut health [77,78]. A more detailed analysis of these active ingredients can improve the formulation of more effective feed additives, enhancing their application as viable alternatives to conventional antibiotics. 

Using these natural antimicrobials also aligns with consumer preferences for more natural and sustainable animal production practices. Among waste or loss with potential for feed applications, grape pomace stands out due to its low cost, abundance, and notably, its bioactive and antibacterial properties, which have garnered increasing research interest [54]. Even if the focus of this section was to underline the antimicrobial effects to support the replace of antibiotics, is should be noticed that the incorporation of plant derived products in animal feed has additional advantages, as they can significantly enhance the quality of animal products. Considering that they are rich in bioactive compounds, such as polyphenols, flavonoids, and essential oils, the nutritional profile of animal feeds can be considerably improved, which in turn, leads to an improve of the overall healthfulness of animal-derived products. Plant extracts can enrich animal products with antioxidants, vitamins, and minerals that are transferred from feed to animal tissues. For example, carotenoids and flavonoids can accumulate in meat, milk, and eggs, enhancing their antioxidant capacity and providing additional health benefits to consumers [79]. This enhanced antioxidant capacity of meat derived products improves their quality by reducing oxidative stress, which in turn helps maintain meat color, flavor, and tenderness. Furthermore, the presence of antioxidants arising from plant extracts prevent lipid oxidation, thus extending the shelf life of meat products and preserving their nutritional value. They also modify the lipid profile of animal products, increasing the content of unsaturated fats (and reducing those of saturated ones), which leads to healthier meat and dairy products. In turn, essential oils can contribute to improve the palatability and reduce off-flavors, masking undesirable flavors in animal feed, thus leading to a higher consumer acceptance and marketability of the final products [80]. Finally, the anti-inflammatory and immunomodulatory properties of these bioactive agents, support animal health by reducing stress and enhancing immune function. Healthier, less-stressed animals produce better-quality meat, milk, and eggs, with lower levels of stress-related hormones that can negatively impact product quality.

Overall, these natural agents with antimicrobial properties also reduce the prevalence of diseases and infections, highlighting their potential as viable alternatives to conventional antibiotics in intensive farming systems [81]. However, their implementation in real livestock remains a significant challenge, especially for Latin American producers, for whom the costs and efficiency should be at least comparable to those of antibiotics. Strong dissemination campaigns targeted at these producers, along with more stringent legislation, could be suitable starting points to address this issue.

### 4.2. Antioxidant Effects

The extraction of different antioxidants and dietary fiber from loss and waste of fruits and vegetables is a subject of extensive investigation. Research indicates that diverse fruit and vegetable co-products, such as citrus peel and pulp, carrot, beetroot, tomato, red beet, grape and others, are rich sources of biologically active substances with proven antioxidant properties (Table 1) [7]. In comparison to edible tissues, peels and seeds contain higher concentrations of phytochemicals, exhibiting greater total phenolic and flavonoid contents than the finished products, with mango seeds and peel demonstrating the highest levels [57]. For this reason, they have been incorporated not only in the formulation of functional foods but also as antioxidants in active packaging [82].

Grape pomace is a co-product of the winemaking process, rich in polyphenols with antioxidant and antimicrobial activities [83]. Oxidative stress may cause a number of pathologies in farm animals that affect the animal welfare and production. When incorporated into the diet of pigs [84] and chickens [85,86,87], grape pomace has a positive effect on their meat by increasing the antioxidant activity in the feed, feces and meat. In fact, the incorporation of grape pomace in chicken diets rich in polyunsaturated fatty acids (more susceptible to oxidative processes) delayed the meat lipid oxidation [88]. Chamorro et al. (2017) found that grape pomace improved the antioxidant status of the animals, increasing the α-tocopherol and reducing the iron content on plasma, not affecting the plasma glutathione [89].

Kaderides et al. (2015) compared the antioxidant capacity of co-products derived from grapes and pomegranate, ascribing the high activity to the presence of phenolic compounds [90]. In the FRAP (ferric reducing antioxidant power) assay, co-products derived from grapes and pomegranate [59] also showed better antioxidant activity than other fruit and vegetable co-products, such as banana peel [91], beetroot pomace [92], and lemon peel [93]. In the ABTS [2,2′-azino-bis(3-ethylbenzothiazoline-6-sulfonic acid) diammonium salt radical cation] assay, pomegranate peel and grape pomace displayed scavenging capacities of 221.5 and 118.7 mg Trolox equivalents per gram of extract dry weight, respectively [94], respectively. These values are much higher than those observed for orange peel, artichoke co-products, beetroot pomace, and tomato peel (28.0, 16.8, 5.1, and 4.2 mg Trolox eq./g extract dry weight, respectively) [92,95].

This antioxidant activity complements the antimicrobial properties of phytochemicals present in fruits and vegetable loss and waste, supporting their use as active ingredients in the formulation of animal feed.

### 4.3. Prebiotic Properties

The phytochemicals occurring in fruit and vegetable co-products (polyphenols, flavonoids) have a demonstrated capacity to enhance the gut health of animals by promoting a balanced intestinal microbiota [96,97,98]. Therefore, this group of compounds can be also considered as prebiotics, that is, substrates that can be selectively utilized by host microorganisms conferring a health benefit [99]. When incorporated into livestock diets, these compounds can selectively stimulate the growth of beneficial gut bacteria (lactobacilli and bifidobacteria), while inhibiting the proliferation of pathogenic bacteria, thereby reducing the incidence of gastrointestinal diseases and the need for antibiotic treatments [76]. This selective modulation of the gut microbiome can lead to enhanced nutrient absorption, reduced gut inflammation, and improved immune function, ultimately promoting better growth performance and productivity in livestock.

The mechanisms behind prebiotic properties include a reduction in oxidative stress and protection of the gut epithelium from damage caused by free radicals [100]. This protection helps maintain the integrity of the intestinal barrier, preventing the translocation of harmful bacteria and toxins into the bloodstream. In addition, the fermentation of fiber (also present in large amounts in fruit and vegetable loss and waste) by gut bacteria produces short-chain fatty acids (acetic, butyric, propionic, lactic), which serve as an energy source for intestinal cells and play a crucial role in maintaining gut homeostasis [99]. The combined prebiotic and antioxidant effects of polyphenols, flavonoids, and dietary fiber not only contribute to a healthier gut environment but also support the overall resilience and productivity of livestock, making them valuable components of animal feed formulations.

Although the prebiotic effect of polyphenols has been strongly demonstrated, it has only recently been investigated, mainly in laboratory animals and for human products [96,97,98]. The potential benefits for animal welfare are significant, but further targeted investigations on animal diets and livestock are still needed.

### 4.4. Non-Nutritional Properties of Phytogenics as Feed Additives

Phytogenics encompass a diverse array of substances categorized based on botanical origin, processing, and composition [101,102]. These feed additives can be classified as herbs (nonwoody flowering plants with medicinal properties), spices (intensely aromatic herbs commonly used in human cuisine), essential oils, aromatic oily liquids extracted from plant materials (e.g., flowers, leaves, fruits, and roots), and oleoresins, extracts derived from plant materials using non-aqueous solvents [103,104]. As feed additives, phytogenic compounds not only preserve flavor but also enhance taste and appearance [105], making them suitable alternatives for developing palatable products with bioactive properties in livestock [75].

## 5. Extraction of Value-Added Ingredients from Fruit and Vegetable Loss and Waste

The variety of bioactive compounds found in fruit and vegetable loss and waste requires specific extraction strategies. To effectively isolate, characterize, and analyze these compounds, it is essential to understand their source and select the most suitable extraction methods for each plant matrix, with environmental impact assessment being a critical aspect to consider [106].

Traditional extraction methods (Soxhlet extraction, maceration, steam distillation) often rely on organic solvents (ethanol, methanol, acetone, hexane), which are particularly effective in isolating polyphenols, flavonoids, and other phytochemicals, with the choice of solvent depending on the polarity of the target compounds. However, this approach requires extended processing times and high temperatures which can alter certain phytochemicals [107,108], along with associated environmental costs [109,110]. This has driven the development of green extraction techniques, which prioritize the use of renewable resources and environmentally friendly solvents that are less toxic and readily biodegradable. This not only contributes to the production of high-value products but also minimizes the environmental footprint of the extraction process.

More sustainable extraction techniques include microwave-assisted extraction, ultrasound-assisted extraction, supercritical fluid extraction, pressurized hot water extraction, pressurized liquid extraction, pulsed electric field-assisted extraction, ohmic heating-assisted extraction, and enzyme-assisted extraction [111,112,113]. These methods offer potential advantages in terms of reduced processing time, lower energy consumption, and improved stability of the phytochemicals.

Microwave and ultrasound-assisted extractions enhance solvent penetration into plant materials by utilizing microwave or ultrasound waves, thereby increasing the yield and efficiency of bioactive compound extraction [109]. These methods are particularly beneficial for extracting heat-sensitive ingredients. Supercritical extraction generally employs supercritical CO_2_ as a solvent, providing a high-efficiency method to extract lipophilic substances (essential oils, carotenoids) without leaving toxic residues [114,115,116,117]. Pressurized hot water extraction utilizes water at elevated temperatures and pressures to extract polar compounds. Under these conditions, the water properties change, allowing it to act as an effective solvent, reducing the need for organic solvents and enhancing the extraction of heat-sensitive compounds. Pressurized liquid extraction (also known as accelerated solvent extraction) employs high pressure and temperature to increase the efficiency of solvent-based extraction. It improves the solubility and diffusion rates of target compounds, allowing for faster and more efficient extraction compared to traditional methods. Pulsed electric field-assisted extraction involves the application of short, high-voltage pulses to plant material. These electric pulses create temporary pores in cell membranes, facilitating the release of intracellular compounds, including phytochemicals, while preserving the structural integrity of heat-sensitive compounds. Ohmic heating-assisted extraction utilizes an electric current passed directly through the plant material, generating heat uniformly and rapidly. This technique enhances the extraction efficiency by disrupting cell walls and membranes, leading to the efficient release of phytochemicals, while minimizing thermal degradation. Finally, enzyme-assisted extraction employs enzymes such as cellulase and pectinase to break down cell walls, thereby facilitating the release of intracellular compounds and obtaining high-quality extracts from fruit and vegetable residues [101,118].

Besides the extraction methods, the stability of the extracted compounds is an equally significant concern that warrants thorough examination. Stability issues, such as degradation of bioactive compounds (polyphenols, carotenoids, essential oils), which leads to the loss of efficacy (antimicrobial, antioxidant, prebiotic properties), and changes in sensory properties, can significantly impact the performance of these additives in animal feed. The main factors leading affecting such stability include light, temperature, oxygen exposure, and decrease of pH. Therefore, addressing these stability challenges involves selecting appropriate extraction methods and storage conditions that preserve the bioactivity of these compounds. Encapsulation techniques (microencapsulation, nanoencapsulation), as well as the use of biopolymer matrices, can enhance the stability of these plant-derived additives, protecting them from environmental stressors and ensuring their efficacy throughout the shelf life of the product [39,101].

The development of green solvent systems, particularly aqueous-based alternatives to conventional organic and inorganic solvents, is a primary focus in extraction research. Simultaneously, non-conventional, non-thermal extraction approaches are being explored to enhance sustainability and reduce energy consumption [105]. In this way, the extraction of phytochemicals from fruit and vegetable loss and waste aligns with environmental sustainability goals [119,120,121].

## 6. Safety of Extracted Value-Added Ingredients

The safety of extracted value-added ingredients from fruit and vegetable loss and waste for animal production is a critical area of concern, encompassing different aspects, such as chemical contaminants, microbial safety, nutritional consistency, and potential anti-nutritional factors. As the agricultural and food industries seek to enhance sustainability and economic efficiency, the use of these ingredients in animal feed presents both opportunities and challenges. Ensuring the safety of these ingredients is paramount to protecting animal health and the integrity of the food supply chain.

Chemical contaminants are a major safety concern when dealing with the loss and waste of fruits and vegetables. Among these contaminants, pesticides used to prevent pests and diseases can persist on the products and, consequently, in the co-products as well [122,123,124]. When these co-products are used in animal feed, pesticide residues can accumulate in animal tissues, posing potential health risks for humans who consume animal-derived food products. Other chemical contaminants include environmental ones, such as heavy metals and polychlorinated biphenyls. These substances can be absorbed by plants from contaminated soil or water and accumulate in co-products [125]. When contaminated co-products are used in animal feed, the toxins can bioaccumulate in animal tissues, potentially entering the human food supply [126]. For this reason, regular monitoring and stringent controls of both pesticides and environmental contaminants in fruit and vegetable loss and waste are essential to prevent the introduction of harmful residues into the food chain [126].

Microbial safety is another critical concern, as fruit and vegetable loss and waste can contain different microorganisms, including pathogens. The warm and moist environments where these co-products are often stored can promote the growth of bacteria, molds, and yeasts. If these microorganisms are not adequately controlled, they can cause diseases in animals, reduce feed efficiency, and potentially transfer pathogens to humans through the consumption of animal products [127]. Ensuring the microbial safety of co-products requires implementing good manufacturing practices, including proper sanitation, storage conditions, and where necessary, treatments such as drying, fermentation, or the use of preservatives to inhibit microbial growth. Another issue related to microbial safety is the presence of mycotoxins (toxic compounds produced by certain molds), which represent a significant concern. Mycotoxins can contaminate fruit and vegetable co-products during growth, harvest, or storage [128,129]. Since they are highly stable, these toxins can persist through processing, posing serious health risks to both animals and humans [130]. Controlling mycotoxins requires regular screening and the implementation of appropriate measures, such as using mold inhibitors and ensuring proper storage conditions. These steps are crucial for safeguarding feed safety.

The nutritional consistency of fruit and vegetable loss and waste is another factor to be considered. As they are co-products, the composition is not standardized but mainly depends on the type of product, the agricultural practices employed and the processing methods. This variability can affect the balance of nutrients in the animal feed product. Therefore, the standardization of the inputs used to formulate feed products that include fruit and vegetable loss and waste is essential to ensure a consistent and reliable nutritional profile [131]. This requires the implementation of routine analytical testing to verify the nutrient content and adjust formulations as needed to meet the dietary requirements of the different animal species.

Anti-nutritional factors present another challenge (Table 1). Some types of fruit and vegetable loss and waste contain compounds that can interfere with the digestion and absorption of nutrients. For example, certain seeds and peels contain tannins, phytates, and oxalates, which can bind to minerals and reduce their bioavailability [132]. These anti-nutritional factors must be identified and mitigated to prevent negative impacts on animal health and productivity. Processing techniques such as heat treatment, fermentation, and enzymatic treatments can help reduce the levels of anti-nutritional compounds, enhancing the safety and nutritional value of the co-products [133].

The economic viability and sustainability of using fruit and vegetable co-products in animal feed depend on effectively addressing safety concerns. Research should focus on optimizing processing methods to improve the safety and nutritional quality of these co-products. Collaboration among industry stakeholders, regulatory agencies, and research institutions is crucial for developing and implementing best practices. Additionally, educating farmers and feed manufacturers about potential risks and safety measures is essential. Training programs and guidelines can help ensure consistent adherence to safety protocols, minimizing contamination risks and protecting both animal health and the food supply chain.

## 7. Enhancing Animal Feed in Latin America with Fruit and Vegetable Loss and Waste

The incorporation of fruit and vegetable loss and waste into feed products in Latin America is a virgin niche with significant opportunities for innovation in agribusiness. The agricultural sector of this region is a cornerstone of many economies, providing a significant portion of both domestic food supplies and export revenues. Countries like Brazil, Argentina, and Mexico have robust agricultural sectors and the necessary infrastructure to process and distribute these co-products effectively. Using fruit and vegetable co-products in animal feed presents a significant opportunity for farmers to save costs by utilizing locally sourced and inexpensive feed ingredients. Moreover, this practice helps reduce the amount of organic waste sent to landfills, where it would otherwise contribute to methane emissions. The benefits of this approach also extend to the economy, as it fosters the development of a co-products market, creating new revenue streams for farmers and food processors and driving innovation in food processing technologies and waste management practices [134].

Agricultural activities in marginal areas of Latin America are of great social importance, as they provide a critical livelihood for local populations. In regions that have experienced significant depopulation in recent decades, agro-pastoral practices often represent one of the few viable economic activities. Moreover, sustainable agricultural practices are vital for preserving local farming traditions and cultural heritage, particularly those related to pastoralism, cheese-making, and other traditional practices [135]. From a social perspective, using co-products in animal feed can enhance food security by efficiently utilizing available resources, making food systems more effective, and potentially reducing the overall cost of food production [136]. This approach can result in lower prices for consumers and increased access to nutritious food products. Moreover, the development of co-product utilization initiatives can create jobs in rural areas, further supporting community development.

Despite all the mentioned benefits, the utilization of the widely available fruit and vegetable co-products in Latin American animal feed remains low, with antibiotics still being prevalent [137]. This reliance on antibiotics, often as growth promoters, underscores a critical issue: the need to shift towards more sustainable and natural feed alternatives. The successful implementation of co-products in animal feed requires overcoming several challenges, with ensuring the safety and quality of these feed ingredients being paramount.

Educating producers on the benefits of these co-products is essential, as is addressing the associated costs and challenges. Commercial strategies to introduce these co-products into the market could include creating certification programs for sustainably produced animal feed, developing partnerships between agricultural producers and feed manufacturers, and launching marketing campaigns that highlight the nutritional and environmental benefits of these feed ingredients. In addition, addressing regulatory gaps regarding antibiotic use is crucial. Implementing stricter regulations and promoting alternatives can drive the adoption of fruit and vegetable co-products. Moreover, government support, in the form of research funding and policy incentives, can further facilitate the adoption of these practices. By emphasizing the importance of incorporating these co-products, educating producers, and developing robust commercial strategies, Latin America can lead the way in sustainable animal production, reducing the environmental footprint and enhancing food safety and quality.

Looking forward, the role of food additives derived from fruit and vegetable loss and waste is expected to expand significantly in the Latin American food industry. As demand grows for natural and sustainable alternatives in both human and animal nutrition, the potential of these additives will be crucial in shaping future food systems. They can contribute to food safety, reduce environmental impacts, and provide health benefits, making them valuable tools for sustainable food production. This shift towards using bioactive food additives is a vital step in developing innovative solutions that address current challenges in food safety and sustainability in Latin America.

## Data Availability

No new data were created or analyzed in this study. Data sharing is not applicable to this article.
